# Limitations of the DiaRem Score in Predicting Remission of Diabetes Following Roux-En-Y Gastric Bypass (RYGB) in an ethnically Diverse Population from a Single Institution in the UK

**DOI:** 10.1007/s11695-016-2368-9

**Published:** 2016-09-10

**Authors:** George Tharakan, Rebecca Scott, Olivia Szepietowski, Alexander D. Miras, Alexandra I. Blakemore, Sanjay Purkayastha, Ahmed Ahmed, Harvinder Chahal, Tricia Tan

**Affiliations:** 1 Department of Medicine, Imperial College London, London, UK; 2 Department of Endocrinology, Imperial College Healthcare NHS Trust, London, UK; 3 Department of Surgery, Imperial College Healthcare NHS Trust, London, UK; 4 Department of Life Sciences, Brunel University, London, UK

**Keywords:** Diabetes, DiaRem, Diabetes remission, RYGB, Bariatric surgery

## Abstract

**Purpose:**

This study aimed to determine the predictive power of the DiaRem score following Roux-en-Y gastric bypass to identify patients who would have diabetes remission at 1 year in an ethnically diverse population.

**Methods:**

We performed a retrospective review of 262 patients with type 2 diabetes mellitus who underwent RYGB at the Imperial Weight Centre, UK, from 2007 to 2014. Data was collected on the parameters required to calculate the DiaRem score as well as pre- and post-surgical weight and the ethnicity of the subjects.

**Results:**

The studied cohort was ethnically diverse (61.3 % Caucasian, 10.3 % Asian, 5.3 % black, 2.6 % mixed and 20.6 % other). At 1-year post-surgery, there were significant reductions in mean weight (133.4 to 94.3 kg) and BMI (46.7 to 33.3 kg/m^2^). The mean HbA1c decreased from 8.2 to 6.1 %, and 32.5 % of the cohort underwent either partial or complete remission. 67.8 % of the patients that were classified in group 1 of the DiaRem score (most likely to have remission) had complete remission. However, 22.9 % of the patients predicted to have the least chance of remission had either partial or complete remission.

**Conclusions:**

In this ethnically diverse cohort, the DiaRem score remains a useful tool to predict diabetes remission in those that have a low DiaRem score (high chance for remission) but was more limited in its predictive power in those with a high DiaRem score (least likely to have remission). Caution must be used in the application of this model in populations other than the US white Caucasian population used to derive the score.

## Introduction

The incidence of type 2 diabetes mellitus (T2DM) continues to rise within the UK, driven by the obesity epidemic [1]. Roux-en-Y gastric bypass (RYGB) surgery is currently the most effective treatment for T2DM resulting in long-term remission in a proportion of patients [2–4]. Whilst considered cost-effective and safe, the availability of the operation is unable to meet the demand due to the rapidly rising prevalence of obesity [5, 6]. Furthermore, although RYGB may result in improvement in obesity comorbidities, not all patients with diabetes undergo remission. In the context of a health service with limited resources, a predictive score for the probability of significant diabetes improvement is required to target surgery only to those most likely to benefit. Current predictive scores include the DiaRem [7] and the ABCD scores [8]. The DiaRem score has been validated in three different populations: USA [7, 9], Taiwan [8] and Brazil [10]. However, no external validation has been performed in the UK population. To address this gap, we performed a retrospective study within a tertiary bariatric service in London.

## Methods

We conducted a retrospective review of the records of patients with both obesity and T2DM who had undergone RYGB surgery at Imperial Weight Centre between 2007 and 2014. Preoperative data was collected to calculate the DiaRem score which requires the following: patient’s age, preoperative HbA1c, preoperative use of insulin and other diabetic medications [7]. In addition, patient demographics including ethnicity were recorded. Ethnic categorization was based on codes devised by the Office for National Statistics. There are five main categories: ‘white’, ‘mixed’, ‘Asian or Asian British’, ‘Black or Black British’ and ‘other ethnic groups’. In this system, subjects who originate from India, Pakistan and Bangladesh are classified as ‘Asian’ whilst patients who identify themselves as Chinese are placed in the ‘other ethnic group’. Postoperative data was collected at 1-year post-surgery to determine remission of diabetes: fasting glucose, glycated haemoglobin (HbA1c) and diabetic medication.

Remission of diabetes was defined as per the American Diabetes Association (ADA) criteria [11]. Complete remission requires a fasting glucose below 5.6 mmol/l and an HbA1c below 6.0 % for 1 year, without the use of any diabetic medication. Partial remission is defined as a fasting glucose between 5.6 and 6.9 mmol/l and an HbA1c between 6.0 and 6.5 % for the duration of 1 year, in the absence of any diabetic medication. The patients were therefore analysed for the presence or absence of diabetes remission at least 12 months after surgery.

Patients were divided into five groups by DiaRem score: group 1 (0–2), group 2 (3–7), group 3 (8–12), group 4 (13–17) and group 5 (18–22). In Still et al. [7], a lower DiaRem score predicted a higher chance of diabetes remission, with 88 % of patients in group 1 having partial or complete remission and only 2 % of patients in group 5 having partial or complete remission.

### Statistical Analysis

Data was analysed using GraphPad Prism 6.0 h (GraphPad, San Diego, CA). Paired student’s *t* tests were used to compare pre- and post-surgical variables within our study population, whilst an unpaired *t* test was used to compare this study population with other validated cohorts. A comparison of medication pre- and post-surgery was performed using a Chi-square test. A Cochran-Armitage test was used to test for significance in the association between trend of diabetes remission and DiaRem score.

## Results

### Patient Characteristics

Two hundred sixty-two patients with T2DM and obesity underwent RYGB surgery at the Imperial Weight Centre between 2007 and 2014. The preoperative demographics are shown in Table 1. There was a higher prevalence of female patients (59.9 %). The mean age (±SD) of the population was 51.0 ± 9.5 years and the mean (±SD) preoperative weight was 133.4 ± 23.4 kg. With reference to glycaemic control, the mean (±SD) preoperative HbA1c was 8.2 ± 1.8 %. The most common ethnicity was Caucasian (61.1 %) followed by Asian (10.3 %). At 1-year post-surgery, there were statistically significant reductions in both weight and HbA1c.

**Table 1:** Characteristics of patients who underwent bariatric surgery at the Imperial College Weight Centre 2007 to 2014

	Pre-surgery	1 year post-surgery	*P* value
Age mean (SD) (range) in year	51 (9.5) (23–73)		
Gender no. (%)			
Male	105 (40.1)		
Female	157 (59.9)		
Ethnicity no. (%)			
Caucasian	160 (61.1)		
Asian	27 (10.3)		
Black	14 (5.3)		
Mixed	7 (2.6)		
Other	54 (20.6)		
Weight mean (SD) (range) in kg	133.4 (23.4) (83–232)	94.3 (20.2) (54.0–181.0)	<0.001
BMI mean (SD) (range) in kg/m^2^	45.3(7.1) (33.5–69.2)	32.9 (5.9) (29.2–52.9)	<0.001
HbA1c mean (SD) (range) in %	8.2 (1.8) (5.1–16.4)	6.1 (1.0) (4.6–10.5)	<0.001
Diabetic medication no. (%)			
Metformin	220 (84.0)	151 (57.6)	<0.0001
Sulphonylurea	58 (22.1)	2 (0.01)	<0.0001
Other insulin-sensitizing agent	58 (22.1)	7 (0.02)	<0.0001
Insulin	77 (29.4)	48 (18.3)	<0.003
DiaRem score no. (%) grouped by score			
0–2	28 (10.7)		
3–7	96 (36.6)		
8–12	61 (23.3)		
13–17	42 (16)		
18–22	35 (13.4)		

### DiaRem Scoring and Diabetes Remission

At 1 year, complete remission was seen in 85 patients (27.9 %) while partial remission was seen in a further 12 (4.6 %). 67.8 % of those with DiaRem score 0–2 had complete or partial remission, 38.5 % of those with score 3–7, 27.9 % of those with score 8–12, 9.5 % in those with score 13–17 and 22.9 % in those with score 18–22 (Table 2).

**Table 2:** Comparison of the DiaRem scores from the bariatric surgery population at Imperial Weight Centre (*P* < 0.0001 for trends in both CR/PR and PR, Cochran-Armitage test) and from Still et al. [7], Aminian et al. [9], Lee et al. [8] and Sampaio-Neto et al. [10]

	Imperial Weight Centre (*N* = 262)		Still et al. (*N* = 690)		Aminian et al. (*N* = 136)	Lee et al. (*N* = 245)	Sampaio-Neto et al. (*N* = 70)
DiaRem score	CR/PR no. (%)	CR no. (%)	CR/PR (%)	CR (%)	CR/PR (%)	CR (%)	CR (%)
0–2	19 (67.8)	19 (67.8)	88	61	86	100.0	45.7
3–7	37 (38.5)	35 (38.5)	64	32	78	85.3	42.8
8–12	17 (27.9)	12 (19.7)	23	10	30	43.5	11.4
13–17	4 (9.5)	3 (7.1)	11	5	27	38.1	0.0
18–22	8 (22.9)	4 (11.4)	2	0	20	27.9	0.0

*Abbreviations*: *CR* complete remission, *PR* partial remission

## Discussion

The DiaRem score is a simple, clinically based scoring system to predict the remission of diabetes following bariatric surgery. However, in our UK-based population, the predictive power of the DiaRem score was reduced (Table 2). Only 68 % of the patients in our cohort with DiaRem score of 0–2 experienced remission (completely or partially) compared to 88 % in the US study [7]. In contrast, at scores of 18–22, 23 % of our patients experienced remission (completely or partially) compared to 2 % in the US study. Validation of the DiaRem score in other populations has revealed similar limitations. Lee et al. studied a Taiwanese population and found also that >25 % of their patients with the highest DiaRem scores experienced complete remission despite being predicted to have no chance of complete remission according to the Still et al. data [8]. A similar finding in another US cohort by Aminian et al. showed that 20 % of patients with DiaRem scores 18–20 had either partial or complete remission (as opposed to 2 % from Still et al.) [9]. In contrast to this, Sampaio-Neto et al., who studied a small Brazilian population, demonstrated a better predictive power at higher DiaRem scores although there was a disparity at the lowest DiaRem scores of 0–2 wherein <50 % experienced complete remission (vs 61 % from Still et al.) [7, 10].

The causes of this disparity between different populations are unclear, and the determination of such causes is beyond the power of a retrospective study. However, it should be noted that in terms of age (mean age 50.5 in Still et al. vs 51.0 years in our study), gender prevalence and BMI (presurgical BMI 48.8 in Still et al. vs 45.3 kg/m^2^ in our study), the population studied by Still et al. was similar [7, 8]. The most striking difference is the wider ethnic diversity in our data, which reflects the London-wide population. Ninety-seven percent of patients studied by Still et al. were white Caucasian whilst this was true for only 61.1 % of this cohort. Whilst this observation can only suggest a possible association, it is not inconceivable that ethnic origin may play a role. Previous studies have demonstrated that ethnicity can affect susceptibility to developing T2DM [10, 12] as well as response to diabetes medication, with Asians (who represented 10 % of our study population) doing worse than Caucasians [7, 13]. Similarly, the difference in DiaRem performance noted in the study of Lee et al. might be explained by the fact that they were studying a Chinese population [8].

However, there may be alternative causes for the differences observed in our study. The DiaRem score is also affected by prescribing practices. In the UK study population, sulphonylureas and insulin-sensitizing drugs are less commonly used than in the US population (22 vs 32 %, respectively, for sulphonylureas; 22 vs 31 % for insulin-sensitizing agents) [7]. Furthermore, based on recent medication surveys, insulin prescription is higher in the UK than in the USA (23.3 vs 8.1 %, respectively) [14, 15]. These differences, driven by national guidelines, drug licensing and historical practice, affect the DiaRem score and, therefore, may limit the applicability of the score in the UK population as well as other populations with prescribing practices at variance to those in the USA.

This study has several limitations that should be noted. Whilst it represents the largest cohort so far used to validate the DiaRem score, the studied population remains too small to support meaningful subgroup analyses of the different ethnic groups. Furthermore, whilst this represents the first validation of DiaRem within the UK population, the subject data came from a small cohort treated at a single institution and it is unclear whether the results are generalizable to the entire British population.

The DiaRem score provides an easy-to-determine score for predicting diabetes remission to aid decision-making for surgery. Whilst predictive scores are more useful in the context of assessing response within a large population, increasing pressures on both finances and resources mandate the need for a clinical test to guide both surgeons and patients. However, in our population, use of the DiaRem score may falsely deter patients with higher scores from surgery that would benefit a significant proportion. These higher scoring DiaRem patients represent a subpopulation who have generally worse or more long-standing diabetes, and so potentially have the most to gain by undergoing surgery.

A better predictive tool is required to assess patients placed in the higher DiaRem score group. One previous criticism of the DiaRem score is that it does not take into account duration of diabetes [7–9]. Duration of T2DM has previously been demonstrated to be a strong risk factor for remission of diabetes as it may be a surrogate for pancreatic function [16]. This variable was not included as part of the Still et al. multivariate analysis, but determining duration of diabetes can be imprecise due to the absence of a systematic screening process in most countries and the vague symptoms (or even absence of symptoms) associated with this condition. In addition, whilst the DiaRem score is simple to calculate, it fails to take directly into account any of the postulated mechanisms of diabetes remission such as elevated gut hormone [17] or bile acid secretion [18]. Future prospective studies could evaluate the predictive power of measurement of pre-surgical levels of these mediators as well as investigate different ethnic groups in larger numbers. A final limitation of any predictive score that uses diabetes remission as an end point is that it overlooks any significant improvement in both HbA1c and reduction in medication. Whilst diabetes remission was observed in only 85 patients in this cohort, there was a large reduction in the burden of medication (Table 2).

## Conclusion

The DiaRem score, a model used to predict diabetes remission following bariatric surgery, is less successful for the purposes of predicting remission in the patients treated in this single bariatric unit within the UK. Specifically, it is less useful at predicting response in those who have higher DiaRem score. This may reflect differences in ethnic diversity in the studied populations, or differences in prescribing between countries.
